# Evaluation of patterns in mandibular fractures among South Indian patients

**DOI:** 10.6026/97320630018566

**Published:** 2022-06-30

**Authors:** Sneha Krishnan, Senthilnathan Periasamy, Murugaiyan Arun

**Affiliations:** 1 Department of Oral and Maxillofacial Surgery, Saveetha Dental College and Hospital, Saveetha Institute of Medical and Technical Science, Saveetha University, Chennai 77, India

**Keywords:** mandible, fractures, adults, Chennai population, orthopantograms, maxillofacial trauma

## Abstract

The objective of this study is to evaluate the predicted mandibular fracture pattern among a sample of patients visiting a dental hospital in Chennai, India based on patient demographics. This retrospective analysis involved 46 patients out of which 39 were male and 7 were female who were referred to the Oral Surgery Clinic, Chennai. The medical records and orthopantograms for these 46 patients who received treatment for fractures of the mandible from June 2019- March 2020 were reviewed. Parameters such as age, gender, pattern of distribution, type of mandibular fracture, combination of the fracture and treatment done, were evaluated and assessed by one examiner and reviewed by 2 independent investigators. Data shows that the angle region to be the most common area to be affected (31.67%), accompanied by parasymphyseal region (28.33%), condylar region (13.33%), dento-alveolar region ( 10%), body (8.33%), symphyseal region ( 6.67%) and finally the coronoid region ( 1.67%). Data analysis also revealed that 84.78% of all patients with fractures of the mandible were male and 34.78% of all patients were in the age group of 21-30 years. Most fractures presented with a single fracture site (60.87%), and among combinations of fractures, fractures of parasymphyseal region along with angle region (41.67%) were seen more commonly. Pearson's Chi Square Test was used to determine the association linking the type of mandibular fracture and treatment modality used and p value was < 0.05, which was considered statistically significant. Thus, the patterns of mandibular fractures delineate a significant occurrence of angle fractures among mandibular fractures, commonly seen along with fractures of parasymphyseal region and occurring with a significant male predilection.

## Background:

The mandible is the largest and most powerful facial bone, yet fractures of the mandible are known to constitute a large portion of maxillofacial trauma (second to nasal bone fractures). Due to its prominence, special mobility, and location, the mandible is an unprotected target for injury. [1] Mandible serves an important role in speech, swallowing, and respiration, and also is an esthetically prominent zone, giving individuals their distinct facial characteristics. [2] It is the only ambulatory bone among various facial bones accounting for the lower third of the height of the face. Mandibular fractures cause functional disabilities along with social and cosmetic morbidities [3,4-5], and they occur twice as often as fractures of the midface [6]. In 1650 BC in an Egyptian Papyrus, mandibular fractures were first described [7]. Mandibular fractures can cause injuries ranging from mild facial and head lacerations to catastrophic closed brain damage [8,9]. Due to varied geographic regions, socioeconomic status, cultures, educational status, population density, and study eras, a mutual agreement describing the most common mandibular fracture pattern has not been reached. There is highly restricted knowledge on the unique pattern or kind of mandibular fractures in Asian nations [10]. Mandibular fractures have a multi-factorial pattern. Fracture sites are determined by both the mode of damage and the structure of the mandible. According to studies, the frequency of mandibular fracture varies depending on the location. In the presence of un-erupted third molars, the angle of the mandible is vulnerable, and the condylar region is vulnerable to head-on impacting damage to the chin [2]. According to the literature, the angle is the most prevalent fracture site [11- 13], however, the body [14], and condylar/sub-condylar [15] areas of the mandible seem to be susceptible to fracture [2]. In developing nations like India, greater rates of road traffic accidents and interpersonal violence are considered causative factors in mandibular fractures, with a higher proportion of young adults suffering these injuries [16]. Previously our team had conducted numerous studies which include in vitro studies [17], reviews [18, 19], surveys [20-23], and clinical trials [24-31]. Therefore, it is of interest to delineate the changing trends of fracture patterns of the mandible based on demographics of the patients among a sample of patients visiting a dental care hospital in Chennai, India.

## Materials and methods:

Our study was conducted during the period between June 2019 and March 2020. 86000 patient records were examined and analyzed in this study. There were 46 patients with radiographically confirmed mandibular fractures, 39 of whom were male and 7 of whom were female.

## Inclusion criteria:

[1] Age group between 8-70 years

[2] Both genders

[3] Patients with ortho pantomographs as records for radiographically confirming mandibular fractures

## Exclusion criteria:

[1] Patients with facial lacerations without fracture

[2] All maxillofacial injuries excluding mandibular fractures

[3] Patients with development disorders, pathology, tumors of the mandible.

## Parameters:

The data extracted for the purpose of the study were as follows:

[1] Age

[2] Gender

[3] Pattern of Distribution of Mandibular Fracture

[4] Type of mandibular fracture

[5] Combination of mandibular fracture

[6] Type of treatment modality done

The findings were thereafter recorded and the subjects were divided into 7 age groups- Group 1: 0-10 years, Group 2: 11-20 years, Group 3: 21-30 years, Group 4: 31-40 years, Group 5: 41-50 years, Group 6: 51-60 years, Group 7: 61-70 years.

## Data collection:

Patients who visited the Outpatient Department of the Oral Surgery Clinic at Saveetha Dental College in Chennai between June 2019 and March 2020 provided data for the study parameters. The work was approved by Saveetha University's Institutional Ethical Committee (SDC/SIHEC/2020/DIASDATA/0619-0320). One examiner completed all of the assessments, and the findings were reviewed and recorded by two independent investigators.

## Statistical analysis:

IBM SPSS version 23.0 software was used to tabulate and analyze the data. The descriptive statistics of frequency and percentage were used to analyze non-parametric data. The connection between the type of mandibular fracture and the treatment modality was determined using Pearson's Chi Square Test.

## Results:

This study included a total of 46 fractures of mandible cases with complete medical and radiological records.

## Age distribution:

The patients with the youngest and oldest ages were 8 and 70 years old, respectively. According to the age distribution of study participants, patients aged 21-30 years suffered the most mandibular fractures (34.78%) as shown in Figure 1(see PDF).

## Gender distribution:

The distribution of study subjects based on gender, over a 10 month period, revealed that 39 patients (84.78%) men and 7 patients (15.22%) women experienced mandibular fractures (Figure 2 - see PDF). Location of Fracture (Site) and anatomy: The distribution of study subjects based on fracture sites showed that out of 46 patients, 28 patients (60.87%) had unilateral fractures (Figure 4 - see PDF). Angle fractures were more commonly seen (31.67%), followed by Para-symphysis fractures (28.33%), Condyle fractures (13.33%), Dento-alveolar fractures (10%), Body fractures (8.33%), Symphysis fractures (6.67%) and lastly coronoid fractures (1.67%) (Figure 3 - see PDF). Out of the 46 patients, 12 patients presented with combination fractures, out of which para-symphysis with angle fractures (41.67%) were most commonly seen, followed by combination of symphysis with condylar fractures (16.67%) Para-symphysis with body fractures, para-symphysis with condylar fractures, para-symphysis with both coronoid and condylar fractures, symphysis with angle fractures and bilateral para-symphysis with angle fractures all yielded the same result (8.33%) (Figure 6 - see PDF). The relation between the type of mandibular fracture and the treatment technique utilized was assessed using Pearson's Chi Square Test, and the p value was less than 0.05, indicating that it was statistically significant (Figure 5 - see PDF).

## Treatment modalities:

78.26% of patients required active treatment, with open reduction and internal fixation (with or without inter-maxillary fixation) being the most common treatment options. In 21.74 percent of cases, conservative therapy was adopted. In 50% of instances, conservative therapy was performed when fractures were localized to a single condyle region. Active treatment was necessary in 14 cases with fractures involving more than one site on the mandible (18 instances) (77.77 percent) (Figure 7 - see PDF). Since the mandible is the only facial bone that is mobile, the fracture of the mandible is never overlooked because it causes excruciating pain that worsens with mastication and phonation movements, as well as respiratory movements [32]. It is critical to comprehend their epidemiology, as this will allow us to better target our prevention efforts and reconstruct present trauma evaluation techniques [33].

## Discussion:

Data on the pattern of mandibular fractures validate known data, particularly in terms of age and gender. This study found that the age group 21-30 years had the highest frequency of mandibular trauma (34.78 percent), which is consistent with research by Subhashraj et al. (28%) [10], Lin et al. (31%) [1] and Samman et al. (57.36%) [34] However, Ogundare et al. [13] found that the age group 25-34 years had the highest frequency of mandibular trauma in their study on fracture patterns on the mandible in an urban trauma center (37.29 percent). The reason behind our study showing a higher incidence among young adults may be due to the fact that the people were engaged in fights, violent physical activities, sports, high-speed vehicles, and lack of safety measures during this phase of life. In our study, there was significant male predilection (84.78%). This is in line with other research done by Ogundare et al. on gender and mandibular trauma [13], Kamulegeya et al. [35], Ahmed et al. [36], Leles et al. [37], Qudah et al. [38]. However, Subhashraj et al. [10] observed a significant female involvement which may be related to change in work culture where men and women are getting equal opportunities [16]. The presentation pattern demonstrated a greater incidence of angle region involvement (31.67%), which is consistent with the findings of Olson et al. [39], Ogundare et al. [13] and Fridrich et al. [40]. Nevertheless, Adi et al. [41], Lin et al. [1], Subhashraj et al. [10] revealed that the symphyseal region is the most prevalent site of mandibular fractures. Most fractures in our study presented as unilateral fractures (60.87%) and this is in contrast to the study performed by Lee et al. [2] who showed a higher occurrence of multiple fractures (37%), and Ogundare et al. who showed that 52.25% of the cases in the mandible had multiple fracture sites [13]. According to the study, conducted by Lee et al. [2], angle and parasymphysis fractures were the most common combination of fractures, which is in agreement with our study. However, according to Ogundare et al. [13], angle-body fractures were more commonly seen among combination fractures in the mandible. Active treatment (open reduction and internal fixation) was the most commonly employed treatment technique in our study, which is consistent with the findings of Subhashraj et al. [10]. When fractures affected more than one site on the mandible, active treatment was necessary in 86 percent of patients, according to Lee et al. [2], which was consistent with our findings (77.77 percent of the time). The aetiology of trauma was not reported in this study, which are important criteria because fracture patterns differ depending on the mechanism of injury. Also, because only a small number of patients were included, concomitant maxillofacial injuries were not recorded; as a result, our findings and observations cannot be generalized. A focus on education and awareness regarding maxillofacial injuries can help in preventing further trauma and also assist in distributing resources for treatment.

## Conclusion:

Multiple fractures are common in mandibular injuries, necessitating surgical intervention. Studies on mandibular injuries are vital to determine the frequency and etiology of these injuries, which represent community trauma patterns, as well as to train health care providers to detect facial trauma and offer the necessary treatment. Thus a significant incidence of angle fractures among mandibular fracture patterns which are seen commonly in combination with fractures of para-symphyseal region, occurring with a significant male predilection is seen.
